# Genomic and phenotypic insights into broad-spectrum anti*-Escherichia coli* activity of *Lactiplantibacillus argentoratensis* and *Weissella cibaria* isolated from swine feces: A sustainable alternative to antibiotics

**DOI:** 10.14202/vetworld.2025.3476-3495

**Published:** 2025-11-23

**Authors:** Rumpa Jutakanoke, Warunya Chakritbudsabong, Songkran Chuakrut, Wongsakorn Phongsopitanun, Rapee Thummeepak, Wuttichai Mhuantong, Jirasin Koonthongkaew, Noppadon Siangpro, Sasitorn Rungarunlert

**Affiliations:** 1 Department of Microbiology and Parasitology, Faculty of Medical Science, Naresuan University, Phitsanulok, 65000, Thailand; 2 Centre of Excellence in Medical Biotechnology, Faculty of Medical Science, Naresuan University, Phitsanulok, 65000, Thailand; 3 Department of Pre-Clinic and Applied Animal Science, Faculty of Veterinary Science, Mahidol University, Nakhon Pathom, 73170, Thailand; 4 Laboratory of Cellular Biomedicine and Veterinary Medicine, Faculty of Veterinary Science, Mahidol University, Nakhon Pathom, 73170, Thailand; 5 Department of Biochemistry and Microbiology, Faculty of Pharmaceutical Sciences, Chulalongkorn University, Bangkok, 10330, Thailand; 6 National Center for Genetic Engineering and Biotechnology (BIOTEC), National Science and Technology Development Agency, Pathum Thani, 12120, Thailand; 7 Department of Microbiology, Faculty of Sciences, Chulalongkorn University, Bangkok, 10330, Thailand; 8 Research Unit in Bioconversion/Bioseparation for Value-Added Chemical Production, Chulalongkorn University, Bangkok, 10330, Thailand; 9 Department of Epidemiology and Public Health Emergency Response, Office of Disease Prevention and Control, Region 3 Nakhon Sawan, Nakhon Sawan, 60000, Thailand

**Keywords:** antimicrobial resistance, *Escherichia coli*, *Lactiplantibacillus argentoratensis*, Probiotics, SDG 12 (responsible consumption and production), SDG 15 (life on land), SDG 2 (zero hunger), SDG 3 (good health and well-being), sustainability, swine health, *Weissella cibaria*

## Abstract

**Background and Aim:**

Antimicrobial resistance (AMR) resulting from antibiotic misuse in livestock poses a growing threat to animal and human health. The development of sustainable probiotic alternatives supports the United Nations Sustainable Development Goals (SDGs) for zero hunger (SDG 2), good health and well-being (SDG 3), and responsible consumption and production (SDG 12). This study aimed to isolate, characterize, and perform genomic analysis of lactic acid bacteria (LAB) from swine feces exhibiting antibacterial activity against pathogenic *Escherichia coli*, to explore their potential as eco-friendly probiotic feed additives.

**Materials and Methods:**

Thirty fecal samples were collected from slaughtered crossbred pigs in Thailand. LAB isolates were screened for antibacterial activity against five *E. coli* pathotypes (Enteroaggregative *E. coli*, enterohemorrhagic *E. coli*, enteroinvasive *E. coli*, enterotoxigenic *E. coli*, and enteropathogenic *E. coli*) and assessed for acid and bile tolerance, adhesion capacity, and gastrointestinal survival. Two promising isolates (ATP111 and ATP210) were subjected to whole-genome sequencing and bioinformatics analyses for genes related to antimicrobial production, stress tolerance, virulence, and AMR.

**Results:**

Among 93 initial isolates, *Lactiplantibacillus argentoratensis* ATP111 and *Weissella cibaria* ATP210 exhibited broad-spectrum inhibition against all *E. coli* pathotypes. Both strains survived under pH 2.5 and 1% bile conditions, showing 74.39% and 66.90% survival, respectively, in simulated gastrointestinal conditions. Genomic analyses revealed the presence of genes encoding bacteriocins, polyketide synthases, terpenes, and multiple stress-response proteins, supporting their resilience and antimicrobial functionality. Importantly, both genomes lacked virulence and *AMR* genes, confirming biosafety for probiotic use.

**Conclusion:**

The integrated phenotypic and genomic evidence positions *L. argentoratensis* ATP111 and *W. cibaria* ATP210 as safe, effective, and sustainable probiotic candidates for swine health management. Their application as antibiotic alternatives aligns with SDG 3 (good health and well-being), SDG 12 (responsible consumption and production), and SDG 15 (life on land), contributing to reduced antibiotic dependence and improved livestock sustainability. Future *in vivo* validation is recommended to confirm efficacy and support global AMR mitigation efforts.

## INTRODUCTION

Swine production is a critical component of both Thailand’s and the global agricultural economy, contributing substantially to food security and the livelihoods of millions of people [1]. In 2022, Thailand produced nearly 10 million fattening pigs [2], reflecting its strong role in the livestock sector. However, the industry faces persistent challenges from infectious diseases, particularly those caused by *Escherichia coli*, which threaten animal welfare, production efficiency, and economic sustainability [3]. *E. coli*, a facultative anaerobic, Gram-negative bacterium of the family *Enterobacteriaceae*, is a common inhabitant of the intestinal tract in animals, including pigs. Nevertheless, pathogenic strains, especially enteropathogenic *E. coli* (EPEC) and enterotoxigenic *E. coli* (ETEC), can cause severe enteric colibacillosis, characterized by diarrhea, dehydration, and high mortality in neonatal and post-weaning piglets [4, 5].

Conventional control measures for swine colibacillosis rely heavily on antibiotics, vaccines, and feed additives [6]. Antimicrobial agents such as β-lactams, fluoroquinolones, sulfonamides, and polymyxins have been widely used both for therapeutic and growth-promoting purposes [6–8]. However, the extensive and often indiscriminate use of antibiotics has accelerated the emergence of antimicrobial resistance (AMR), posing a significant threat to animal and public health [9–11]. In Thailand, a high prevalence of multidrug-resistant *E. coli*, including strains carrying colistin-resistance genes (*mcr*-1 and *mcr*-3), has been documented in swine populations [12], underscoring the urgent need for sustainable alternatives to antibiotics. The global AMR crisis aligns with the Sustainable Development Goals (SDGs), particularly SDG 3 (good health and well-being) and SDG 12 (responsible consumption and production), which emphasize reducing antibiotic dependency and promoting sustainable livestock systems [13].

In this context, probiotics have emerged as promising biotherapeutic agents for improving gut health and mitigating pathogen colonization without contributing to AMR. Probiotics are defined as live microorganisms that confer health benefits to the host when administered in adequate amounts. Among them, lactic acid bacteria (LAB) represent the most widely studied group, known for their safety, metabolic versatility, and capacity to produce organic acids and bacteriocins that inhibit pathogens [14–17]. In swine production, LAB supplementation enhances growth performance, immune modulation, and intestinal health, offering a viable alternative to antibiotic growth promoters [18,19].

Autochthonous LAB strains isolated from healthy swine feces are particularly valuable for probiotic development due to their natural adaptation to the swine gastrointestinal environment, competitiveness against intestinal pathogens, and ecological safety [15, 20].

Despite growing recognition of LAB as potential alternatives to antibiotics, comprehensive studies integrating both phenotypic and genomic evidence of probiotic potential from swine-derived LAB remain limited. Most previous investigations have focused on traditional *Lactobacillus* species from food or human sources, while reports on host-specific probiotic strains from healthy swine are scarce. Furthermore, although *Weissella* and *Lactiplantibacillus* species have shown promising antimicrobial and stress tolerance properties, their detailed genomic attributes, such as genes encoding bacteriocins, stress-response systems, and safety determinants, have not been adequately characterized. In particular, the probiotic and anti-*E. coli* activities of *Lactiplantibacillus argentoratensis* and *Weissella cibaria* isolated from swine feces have not been documented in the scientific literature. This lack of integrated genomic and functional validation restricts the rational selection of host-adapted probiotic candidates for livestock application. Addressing this gap is critical for developing effective and safe alternatives to antibiotics in the swine industry, contributing to AMR mitigation and sustainable animal production in line with the SDGs (2, 3, 12, and 15).

This study aimed to isolate, characterize, and perform genomic analysis of LAB strains with antibacterial activity against pathogenic *E. coli* from swine feces in Thailand. The specific objectives were to:

1. Isolate and screen LAB strains for *in vitro* antibacterial activity against major *E. coli* pathotypes causing enteric colibacillosis in pigs.
2. Evaluate probiotic attributes, including acid and bile tolerance, adhesion capacity, hemolytic activity, and survival under simulated gastrointestinal conditions.
3. Conduct whole-genome sequencing and bioinformatics analysis of promising isolates to identify genes associated with antimicrobial production, stress adaptation, virulence, and antibiotic resistance.
4. Correlate genomic features with phenotypic probiotic traits to validate their safety and functional potential.

Through this integrated approach, the study sought to identify safe, host-adapted LAB strains with broad-spectrum anti-*E. coli* activity, specifically *L. argentoratensis* ATP111 and *W. cibaria* ATP210, as potential probiotic feed additives for improving swine gut health and reducing antibiotic dependence in animal production systems.

## MATERIALS AND METHODS

### Ethical approval

The protocol for this study, utilizing fecal samples collected as detailed in the *Sample collection* section from pigs already processed for commercial food production, was evaluated by the Institutional Animal Care and Use Committee of the Faculty of Veterinary Science, Mahidol University. The committee reviewed the study protocol and confirmed that no direct experimentation was performed on live animals. Because the study exclusively used postmortem fecal samples from animals designated for slaughter for commercial purposes, the IACUC concluded that no formal animal ethics approval was necessary. This aligns with ethical standards for research that does not involve direct experimentation or impact on the welfare of live animals.

### Study period and location

The study was conducted from June 2022 to December 2023. The screening and other procedures were conducted at the Department of Microbiology and Parasitology, Faculty of Medical Science, Naresuan University. The whole genome analysis was carried out at the Omics Sciences and Bioinformatics Center, Faculty of Science, Chulalongkorn University.

### Sample collection and storage

A total of 30 fecal samples were collected from the large intestines of individual crossbred pigs (*Sus scrofa*; landrace × large white × Duroc). These samples, serving as the 30 biological replicates for this study, were obtained from two commercial swine slaughterhouses in the provinces of Nakhon Pathom and Pathum Thani, Thailand. These slaughterhouses were certified for good manufacturing practices. All fecal samples were immediately transported to the laboratory in chilled ice packs. The samples were stored at −80°C in tryptone soya broth (TSB) (Cat. No. M011, HiMedia, Thane, India) supplemented with 30% (v/v) glycerol as a cryoprotective agent for long-term preservation.

### Isolation of LAB

LAB was isolated based on the method described by Siangpro *et al*. [21]. Briefly, 5 g of each fecal sample was homogenized with 45 mL of 0.85% (w/v) NaCl solution using a stomacher (HG400vW, WIGGENS, Germany) for 1 min to achieve a 10^−1^ dilution. Subsequently, a 10-fold serial dilution was performed, yielding dilutions up to 10^−8^. Aliquots from the 10^−6^, 10^−7^, and 10^−8^ dilutions were spread-plated onto de Man, Rogosa and Sharpe (MRS) agar (Cat. No. GM641, HiMedia) supplemented with 0.004% (w/v) bromocresol purple. The inoculated plates were anaerobically incubated at 37°C for 48 h. Anaerobic conditions were maintained using an anaerobic jar with a gas generation system (Anaerobentopf 2.5 L, Millipore, Merck, USA). After incubation, individual colonies exhibiting yellow zones, indicative of acid production, were selected and purified by repeated subculturing on MRS agar. Purified isolates were subsequently subjected to a catalase test to confirm their presumptive identification as LAB. The cellular morphology of the bacterial isolates was examined using Gram staining.

### Pathogenic strains of *E. coli* and culture conditions

The pathogenic bacteria used in this study included enteroaggregative *E. coli* DMST 68995 (EAEC), enterohemorrhagic *E. coli* DMST 30544 (EHEC), enteroinvasive *E. coli* DMST 26451 (EIEC), ETEC *E. coli* DMST 30537 (ETEC), and EPEC *E. coli* DMST 30546 (EPEC). All strains were purchased from the National Institutes of Health of Thailand (Department of Medical Sciences, DMST, Thailand). Each strain was cultured in 5 mL TSB at 37°C for 24 h and then diluted to a turbidity of 0.5 McFarland standard. The bacterial suspensions were swabbed onto the surface of Mueller–Hinton agar (MHA) (Cat. No. M173, HiMedia) plates after the incubation.

### Antimicrobial activity screening against pathogenic *E. coli* strains

The antibacterial activity of LAB isolates was evaluated using the agar slab method, as described by Yamashita *et al*. [22]. LAB cells were suspended in a sterile solution to achieve a cell density equivalent to that of the 0.5 McFarland standard. The LAB suspension was then uniformly spread onto the surface of MRS agar plates and incubated anaerobically at 37°C for 24 h. After incubation, 6-mL slabs were aseptically cut from the LAB culture plates (Cat. No. K1004, Hycon, Bangkok, Thailand). These slabs were transferred onto the surface of MHA plates that had been previously inoculated with the pathogenic bacterial strain. The plates were anaerobically incubated at 37°C for 24 h. Following incubation, the diameter of the resulting inhibition zone was measured using a Vernier caliper (Series 230, Mitutoyo, Japan); an inhibition zone diameter <7 mm was considered as no activity, ≥7 mm was considered as activity [23]. Only isolates with activity were considered for further analysis. For negative controls, sterile MRS agar plates without microbial growth were used, while a 15-μg clarithromycin antibiotic disk served as the positive control.

### Functional LAB characterization

#### Acid tolerance test

The resistance of the LAB isolates exhibiting antibacterial activity to low pH was evaluated using a previously described protocol by Siangpro *et al*. [21]. Briefly, 12-h LAB cultures grown under anaerobic conditions were adjusted to a turbidity of 0.5 McFarland standard. Bacterial cells were harvested by centrifugation at 4,000 × *g* for 10 min and resuspended in sterile 0.85% (w/v) NaCl solution. An aliquot of 100 μL of the cell suspension was subsequently inoculated into 5 mL of MRS broth acidified to pH 2.5 using 1 M HCl. The inoculated broth was anaerobically incubated at 37°C for 6 h. The LAB growth was quantified by measuring the optical density (OD) at 640 nm after 6 h using a spectrophotometer (SP-880, Metertech, Taiwan). The OD_640_ values were compared with those of the negative control group, in which LAB isolates were inoculated into MRS broth at a normal pH. An increase in OD_640_ in the acidified broth was considered indicative of bacterial growth under low pH conditions.

#### Bile salt tolerance test

Overnight cultures of LAB isolates were adjusted to a turbidity of 0.5 McFarland standard. Bacterial cells were harvested by centrifugation at 4,000 × *g* for 10 min. The resulting cell pellets were resuspended in 0.85% (w/v) sterile NaCl solution. Following resuspension, an aliquot of 100 μL of the bacterial suspension was added to 5 mL of MRS broth supplemented with 1% (w/v) bile salt (Cat. No. RM008, HiMedia). Cultures were inoculated under anaerobic conditions at 37°C for 4 h. Bacterial growth was quantified by measuring the OD at 640 nm using a spectrophotometer. The OD_640_ values of the LAB isolates cultured in the presence of bile salt were compared with those of the negative control cultures grown in MRS broth without the addition of bile salt.

#### Adhesion property test

The ability of LAB to adhere to abiotic surfaces was assessed following the protocol as described by Siangpro *et al*. [21]. Briefly, LAB cultures were grown anaerobically in MRS broth at 37°C for 24 h. Subsequently, all cultures were diluted 100-fold with fresh sterile MRS broth and inoculated into 16 × 100 mm glass test tubes containing the respective broth medium. The negative control test tubes contained only sterile MRS broth. All cultures were incubated under the same anaerobic conditions for 24 h at 37°C. The culture medium was carefully aspirated after incubation. The test tubes were washed twice with phosphate-buffered saline (PBS; pH 7.4) (Cat. No. 18912-014, Gibco, New York, USA) by gentle rinsing to remove non-adherent bacterial cells. The test tubes were then inverted and allowed to air-dry for approximately 1 h. Subsequently, 5 mL of 1% (w/v) crystal violet solution (Cat. No. 94448, Sigma-Aldrich, USA) was added to each tube for 5 min at room temperature to stain the adherent cells. The crystal violet solution was carefully removed, and the test tubes were rinsed thoroughly 3 times with sterile distilled water. The crystal violet bound to the attached bacterial cells on the inner surfaces of the test tubes was solubilized by adding 5 mL of 95% (v/v) ethanol. The extent of bacterial adhesion was determined by measuring the absorbance at 570 nm (Abs_570_) using a spectrophotometer.

#### Hemolytic activity test

To evaluate the safety of the LAB isolates, the hemolytic activity was screened. Overnight cultures of each LAB isolate were streaked onto the surface of TSA containing 5% (v/v) sheep blood. Following anaerobic incubation at 37°C for 24 h, hemolysis surrounding the bacterial colonies was examined on the plates. *Staphylococcus aureus* (ATCC 25923) and *E. coli* (ATCC 25922) were used as positive and negative controls, respectively. The sheep blood agar plates were visually inspected for evidence of β-hemolysis (characterized by clear zones around the colonies), α-hemolysis (characterized by green-hued zones surrounding the colonies), or γ-hemolysis (defined by the absence of any hemolytic zones around the colonies). This experiment was conducted in triplicate on three independent occasions.

#### Resistance to simulated gastrointestinal conditions

The resistance of the LAB isolates to simulated gastrointestinal tract conditions was determined using a previously described protocol by Dawangpa *et al*. [13]. Freshly cultured LAB cells were harvested by centrifugation at 7,000 × *g* for 5 min at 4 °C. The cell pellet was washed twice with sterile 0.85% (w/v) NaCl. The cells were resuspended in sterile simulated gastric juice (PBS, pH 2.0, containing 3 mg/mL pepsin [Cat. No. 417071000, Thermo Fisher Scientific, USA]). The resulting suspensions were incubated for 3 h at 37°C. After incubation, the cells were collected by centrifugation, and the number of surviving cells was determined. LAB cells were harvested and washed as described above and resuspended in sterile simulated intestinal juice (PBS, pH 8.0, containing 3 mg/mL pancreatin (Cat. No. P0636, TCI, Japan) and 1% (w/v) bile salt) to assess resistance to simulated intestinal juice. The suspensions were incubated at 37°C for 4 h. As a negative control, a parallel suspension of cells was incubated under the same conditions (37°C for a total of 7 h) but in neutral PBS (pH 7.0) without the addition of digestive enzymes or bile salts. At the end of the respective incubation periods (3 h for gastric juice and 4 h for intestinal juice), a 0.1-mL aliquot of each isolate suspension was removed, and the number of viable cells was counted by spread plating on MRS agar. The survival rate was calculated using the following equation:


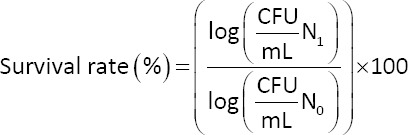


Where N_1_ represents the total viable count of LAB isolates (CFU/mL) following exposure to either simulated gastrointestinal tract fluid or PBS at normal pH (control) for 7 h, and N_0_ represents the initial total viable count of LAB isolates (CFU/mL) before treatment.

### Molecular identification

#### 16S ribosomal RNA sequencing

Genomic DNA was extracted and purified from the overnight cultures of the selected LAB isolates. The 16S *rRNA* gene was amplified using PCR with the universal primers 27F (5′-AGA GTT TGA TCC TGG CTC A-3′) and 1492R (5′-GGT TAC CTT GTT ACG ACT T-3′) [14]. PCR amplification was performed using a Biometra TAdvanced Thermal Cycler (Analytik Jena, Germany). Successful amplification of the 16S *rRNA* gene was confirmed by electrophoretic separation of PCR products on a 1.0% (w/v) agarose gel (Cat. No. D0012, Biobasic, Ontario, Canada) stained with ethidium bromide. The separated DNA fragments were visualized under ultraviolet transillumination (Cleaver Scientific, UK), and their size was estimated by comparison with a DNA ladder. For DNA sequencing, PCR amplicons exhibiting the expected size of approximately 1.5 kb were submitted to Macrogen (Seoul, South Korea).

#### Phylogenetic analysis

The resulting 16S *rRNA* gene sequences were aligned and assembled into complete sequences using the BioEdit software (version 7.2.5; https://bioedit.software.informer.com). The closest relatives were identified by conducting a Basic Local Alignment Search tool (BLAST) [https://blast.ncbi.nlm.nih.gov/Blast.cgi] search against the EzBioCloud database [https://www.ezbiocloud.net] [24]. A phylogenetic tree was constructed based on the neighbor-joining method using the MEGA software (version 7.0) [https://www.megasoftware.net]. Before the analysis, gaps and missing data were removed. The reliability of the phylogenetic tree was assessed using bootstrap analysis with 1,000 replicates [25].

### Whole-genome sequencing and bioinformatics analysis (WGS)

#### DNA extraction and quality control

Genomic DNA was extracted from the two selected isolates using the Wizard Genomic DNA Purification Kit (Promega Corporation, USA) according to the manufacturer’s instructions. The extracted DNA was quantified and its quality assessed using an Agilent 2100 Bioanalyzer system (Agilent Technologies, USA).

#### Library preparation and sequencing platform development

The DNA libraries for each genomic DNA sample were prepared following the Nextera XT DNA Library Prep Kit Reference Guide (Illumina Inc., USA). The resulting two libraries were subjected to paired-end sequencing on a MiSeq platform following the manufacturer’s protocols (Illumina Inc., USA). The raw sequencing reads from each isolate were initially processed using TrimGalore (version 0.6.7) to remove their barcode and adapter sequences [https://github.com/FelixKrueger/TrimGalore].

#### Assembly and polishing software

*De novo* genome assembly was performed using Unicycler (version 0.4.8; https://github.com/rrwick/Unicycler) [26] using default parameters, and the assembled contigs were subsequently polished using Pilon (version 1.23; https://github.com/broadinstitute/pilon) [27].

#### Functional annotation and databases

Gene prediction and functional annotation of the polished genomes were conducted using the Rapid Annotation using Subsystem Technology server (https://rast.nmpdr.org). Species identification was achieved by calculating the percentage of identical sequences and the number of single-nucleotide polymorphisms (SNPs) between the genomes of the studied isolates and those of established strains. The pairwise average nucleotide identity values were calculated using the BLAST algorithm (ANIb) and the MUMmer algorithm (ANIm) as implemented in JSpeciesWS [28]. Whole-genome SNP (wgSNP) analysis was performed to determine pairwise SNP distances between genome sequences using snp-dists (version 0.8.0; https://github.com/tseemann/snp-dists). AMR and virulence genes were predicted using ABRicate (v0.8) [https://github.com/tseemann/abricate], which was employed to screen the genome assemblies against four publicly available databases: The NCBI Bacterial AMR Reference Gene Database [https://www.ncbi.nlm.nih.gov/pathogens/refgene], the Comprehensive Antibiotic Resistance Database (CARD) [https://card.mcmaster.ca], the ResFinder database, and the Antibiotic Resistance Gene-ANNOTation database (ARG-ANNOT) [https://ardb.cbcb.umd.edu]. Virulence factors were identified using the VFanalyzer tool by searching against the Virulence Factor Database (VFDB) [http://www.mgc.ac.cn/VFs]. The presence of secondary metabolite gene clusters was predicted using antiSMASH (version 5.1.0; https://antismash.secondarymetabolites.org) [29].

### Statistical analysis

All experimental procedures were performed in triplicate (n = 3), and the results are presented as the mean ± standard deviation. Statistical analysis was performed to determine significant differences among the experimental groups. Before statistical analysis, all bacterial enumeration data (Colony forming units/mL) were log_10_ transformed to ensure normal distribution and homogeneity of variance. The transformed data were then evaluated to ensure that they met the parametric testing assumptions. Normality of data distribution was assessed using the Shapiro–Wilk test, and Levene’s test was used to confirm the homogeneity of variance between groups. Parametric tests were employed because the data met these assumptions (p > 0.05). For comparisons involving two groups, independent samples t-tests were used. For comparisons involving more than two groups, one-way analysis of variance was used. Where analysis of variance indicated significant differences, *post hoc* pairwise comparisons were conducted using Tukey’s honestly significant difference test to identify specific differences in the mean OD_640_ values between the low pH and bile salt tolerance treatment groups and the respective control groups. The significance level for all statistical tests was set at p < 0.05. All statistical analyses were performed using the Statistical Package for the Social Sciences software (version 25.0, IBM Corp., Armonk, NY, USA).

## RESULTS

### Isolation and preliminary identification of LAB from swine feces

A total of 93 bacterial isolates were recovered from swine feces and characterized by the formation of a yellow halo around colonies on MRS agar supplemented with bromocresol purple. Of these, 64 isolates were presumptively identified as LAB based on the following phenotypic characteristics: Gram-positive staining, negative catalase reaction, and absence of spore formation. The 64 presumptive LAB isolates exhibited coccoid, bacillary, and diplobacillary morphologies.

#### *In vitro* antibacterial activity of LAB isolates against pathogenic *E. coli*

Among the 64 presumptive LAB isolates, nine demonstrated inhibitory activity against at least one of the tested pathogenic *E. coli* strains. Strains ATP111 and ATP210 exhibited broad-spectrum inhibitory effects, suppressing the growth of all five pathogenic *E. coli* strains tested (EAEC, EHEC, EIEC, ETEC, and EPEC), as evidenced by the formation of distinct inhibition zones as detailed in Table 1. Isolate ATPM192 demonstrated inhibitory activity against *E. coli* strains EIEC and ETEC. The remaining six isolates (ATP10, ATP65, ATPM2, ATPM18, ATPM22, and ATPM282) inhibited the growth of one *E. coli* strain. Table 1 shows the diameters of the inhibition zones produced by these nine isolates against the pathogenic *E. coli* strains.

**Table 1:** Antibacterial properties of lactic acid bacteria isolates against pathogenic *Escherichia coli* using agar slab assay inhibition zone diameters (mm) are shown as the mean ± standard deviation from three independent replicates (n = 3).

Isolates	Inhibition zone (mm)				

	EAEC	EHEC	EIEC	EPEC	ETEC
Positive control	18.00 ± 0.50	12.83 ± 0.76	23.67 ± 1.53	13.00 ± 0.50	19.50 ± 0.50
Negative control	–	–	–	–	–
ATP10	7.17 ± 0.29	–	–	–	–
ATP65	–	–	7.50 ± 0.00	–	–
ATP111	9.33 ± 0.76	9.17 ± 0.29	10.33 ± 0.29	10.00 ± 0.50	10.17 ± 1.04
ATP210	9.83 ± 0.29	10.33 ± 0.29	9.67 ± 0.29	10.50 ± 0.00	11.33 ± 0.29
ATPM2	–	–	7.33 ± 0.29	–	–
ATPM18	7.50 ± 0.00	–	–	–	–
ATPM22	–	–	–	7.67 ± 0.29	–
ATPM192	–	–	7.67 ± 0.58	–	8.00 ± 1.00
ATPM282	–	–	–	8.33 ± 0.58	–

– = No inhibition zone, EAEC = Enteroaggregative *Escherichia coli*, EHEC = Enterohemorrhagic *Escherichia coli*, EIEC = Enteroinvasive *Escherichia coli*, ETEC = Enterotoxigenic *Escherichia coli*, and EPEC = Enteropathogenic *Escherichia coli.*

### *In vitro* evaluation of the probiotic properties of selected LAB isolates

#### Acid and bile salt tolerance

Based on their broad-spectrum inhibitory activity, two LAB isolates, ATP111 and ATP210, were selected for further *in vitro* evaluation of their potential probiotic properties. The survival of the selected isolates was assessed under simulated low pH conditions and in the presence of bile salts (Table 2). In the acid tolerance assay, ATP111 exhibited a significant difference in OD_640_ from 2.400 ± 0.156 in the control group (normal pH) to 0.040 ± 0.014 in the treatment group (pH 2.5). Similarly, ATP210 showed a significant divergence in OD_640_ from 1.805 ± 0.039 in the control group to 0.066 ± 0.002 in the treatment group.

**Table 2:** Effect of low pH and bile salt on the growth of selected lactic acid bacteria isolates. The conditions tested were pH 2.5 for 6 h and 1% (w/v) bile salt for 4 h. The control groups were grown in standard de Man, Rogosa and Sharpe (MRS) broth without the added stressor. Data represent the final optical density (OD_640_) after incubation. Values are the mean ± standard deviation of three independent replicates (n = 3).

Isolates	OD_640_ of the acid tolerance test		OD_640_ of the bile salt tolerance test	
	
	Control group	Treatment group	Control group	Treatment group
ATP111	2.400 ± 0.156^A^	0.040 ± 0.014^B^	2.400 ± 0.156^a^	0.392 ± 0.088^b^
ATP210	1.805 ± 0.039^A^	0.066 ± 0.002^B^	1.805 ± 0.039^a^	0.336 ± 0.059^b^

Means within the same row with different uppercase letters (A, B) represent statistically significant differences (p < 0.05) for the acid tolerance test. Means within the same row with different lowercase letters (a, b) represent statistically significant differences (p < 0.05) for the bile salt tolerance test.

For the bile salt tolerance test, ATP111 significantly decreased OD_640_ from 2.400 ± 0.156 in the control group to 0.392 ± 0.088 in the treatment group (1% bile salt). ATP210 also significantly reduced OD_640_ from 1.805 ± 0.039 in the control group to 0.336 ± 0.059 in the treatment group. Despite this significant reduction in viable cell count, both isolates exhibited tolerance to low pH and bile salt concentrations (Table 2). Measuring OD is a rapid and commonly used method to assess cell growth or survival after exposure to acid or bile salt stress. Many studies have measured the OD after the incubation period to calculate the cell increase or reduction [21, 30, 31].

#### Adhesion and hemolytic activity

The adhesion capabilities of ATP111 and ATP210 to abiotic surfaces were quantified by measuring the absorbance at 570 nm. The absorbance values were 0.198 ± 0.039 for ATP111 and 0.715 ± 0.105 for ATP210. The hemolytic activity of the selected LAB isolates was evaluated for safety. Both ATP111 and ATP210 exhibited α-hemolytic activity when cultured on blood agar, characterized by a greenish halo surrounding the colonies.

#### Survival under simulated gastrointestinal conditions

**Figure 1:**
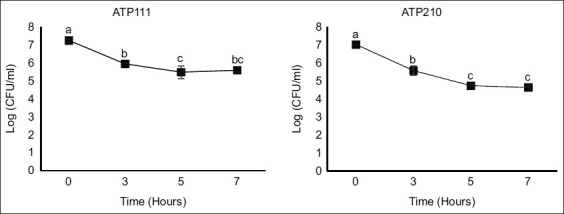
Survival of selected lactic acid bacteria isolates under gastrointestinal tract conditions. The isolates were exposed to simulated gastric juice (pH 2.0) for 3 h and then to simulated intestinal juice (pH 8.0) for 4 h. Viable cell counts were determined by serial dilution and spread plating on de Man, Rogosa and Sharpe agar. Data points are presented as the mean ± standard deviation from three independent replicates (n = 3). Means with different lowercase letters represent statistically significant differences (p < 0.05).

The survival kinetics of isolates ATP111 and ATP210 under simulated gastric and intestinal fluids over 7 h were investigated (Figure 1). Both isolates were initially exposed to simulated gastric juice (pH 2.0) for 3 h, followed by incubation in simulated intestinal juice (pH 8.0) for 4 h. Both isolates exhibited a decline in viable cell counts over time, indicating that the environment was stressful. However, a substantial population of both isolates remained viable at the end of the experiment. The calculated survival rates were 74.39% and 66.90% for ATP111 and ATP210, respectively. ATP111 showed a slightly higher overall survival rate than ATP210 throughout the 7-h exposure.

### Molecular identification of selected LAB isolates

Based on their demonstrated broad-spectrum antibacterial activity and promising *in vitro* probiotic properties, ATP111 and ATP210 isolates were selected for species identification through 16S *rRNA* gene sequencing. The resulting sequences were compared against the EzBioCloud database.

Initial comparison revealed that isolate ATP111 (GenBank accession no. LC764705) showed the highest 16S *rRNA* gene sequence similarity (99.93%) to *Lactiplantibacillus pentosus* DSM 20314^T^ (GenBank accession no. AZCU01000047), while isolate ATP210 (GenBank accession no. LC764706) showed the greatest homology (99.86%) to *W. cibaria* KACC11862^T^ (GenBank accession no. AEKT01000037).

**Figure 2:**
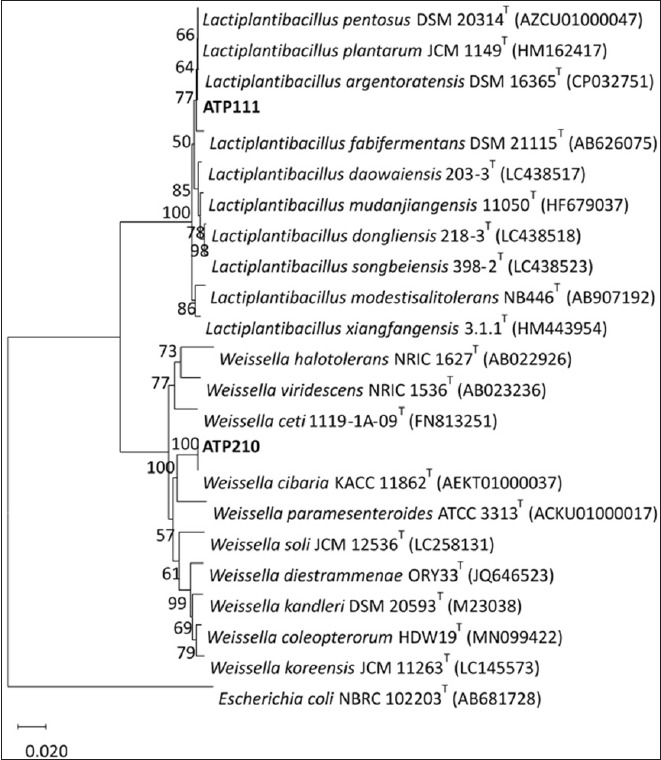
Neighbor-joining phylogenetic tree based on the *16S ribosomal RNA* gene sequences. Bootstrap values (expressed as percentages) from 1,000 replicates are indicated at the nodes.

A neighbor-joining phylogenetic tree was constructed based on the 16S *rRNA* gene sequences of isolates ATP111, ATP210, and closely related type strains for more definitive species identification and to clarify phylogenetic relationships (Figure 2). In this tree, isolate ATP111 formed a distinct cluster with *L. argentoratensis* DSM 16365^T^, supported by a bootstrap value of 77%. This strong phylogenetic affiliation distinguishes it from other closely related species, such as *L. plantarum* and *L. pentosus*, which formed separate branches within the *Lactiplantibacillus* clade, indicating that isolate ATP111 is *L. argentoratensis*.

In the same phylogenetic analysis, isolate ATP210 clustered robustly within the *Weissella* genus, exhibiting a close phylogenetic relationship with *W. cibaria* KACC 11862^T^. This specific clustering was supported by a high bootstrap value (e.g., >95%; please insert the actual value from Figure 2), confirming the identification of ATP210 as *W. cibaria*. Other *Weissella* species, such as *Weissella ceti*, *Weissella viridescens*, and *Weissella halotolerans*, formed distinct separate branches within the tree, further clarifying these classifications. A high percentage of sequence identity in the 16S *rRNA* gene indicated a close phylogenetic relationship at the species level.

### General genomic features of isolates ATP111 and ATP210

The results of the de novo genome assembly for ATP111 and ATP210 isolates are summarized in Table 3. The genome of ATP111 was approximately 3.0 Mbp in size, consisting of 32 contigs, with a gas chromatography (GC) content of 45.2%. In contrast, the ATP210 genome was approximately 2.4 Mbp in size, comprising 23 contigs, and displayed a GC content of 44.9%. Genome annotations of the assembled sequences revealed that ATP111 contained 52 *RNA* genes and 2,980 coding sequences (CDSs), whereas ATP210 contained 72 *RNA* genes and 2,299 CDSs.

**Table 3:** Basic features and statistics of assembled ATP111 (GenBank accession no. GCA_030247085.1) and ATP210 (GenBank accession no. GCA_030247095.1) genomes.

Characteristics	ATP111	ATP210
Total genome size (bp)	3,061,517	2,439,530
Genome coverage	564×	545×
GC-content (%)	45.2	44.9
Number of assembled contigs	32	23
N50	234,863	285,819
Largest contig size (bps)	302,136	660,262
Average contig length (bps)	62,618	20,962
Shortest contig size (bps)	451	320
No. RNAs	52	72
No. coding sequences	2,980	2,299
Genome completeness (%)	99.35	98.77
Contamination (%)	1.39	0.65

The raw sequencing reads and assembled genomes for *L. argentoratensis* ATP111 and *W. cibaria* ATP210 have been deposited in the National Center for Biotechnology Information under BioProject PRJNA957133. The raw Illumina sequencing reads for this project have been deposited in the Sequence Read Archive under accession nos. SRX20019082 (strain ATP111) and SRX20019083 (strain ATP210). The final assembled and annotated genomes are available in GenBank under accession numbers GCA_030247085.1 (strain ATP111) and GCA_030247095.1 (strain ATP210). The whole-genome shotgun sequencing projects have been deposited at GenBank under accession numbers JARXZI000000000 (strain ATP111) and JARXZH000000000 (strain ATP210).

### Whole-genome sequencing-based species identification

**Figure 3:**
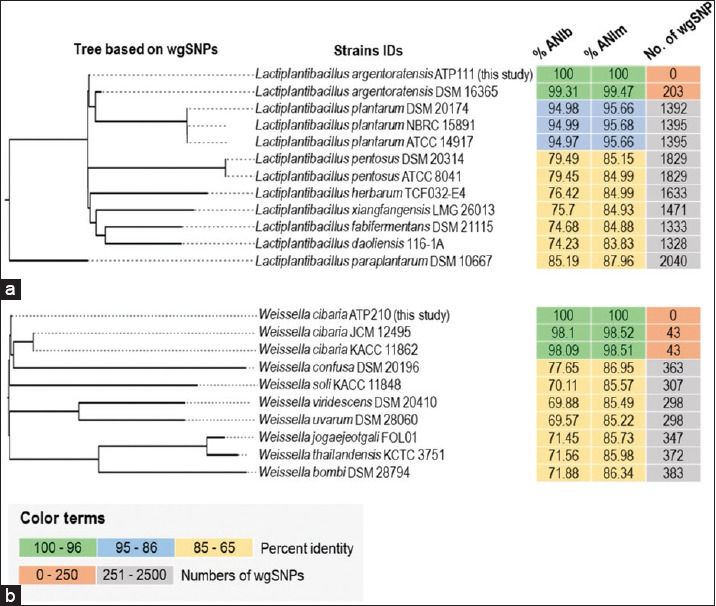
Whole-genome-based identification of isolates ATP111 and ATP210. (a) Demonstration of whole-genome average nucleotide identity (ANI) and whole-genome single-nucleotide polymorphism (wgSNP) distance between *Lactiplantibacillus argentoratensis* ATP111 and the type strain genomes of eight species within the genus *Lactiplantibacillus*. (b) Binary comparison matrix depicting ANI and wgSNP distance between *Weissella cibaria* ATP210 and representative type strains of eight species within the genus *Weissella*.

The species identification of isolates ATP111 and ATP210 was further substantiated using two genomic approaches: ANI values (ANIb and ANIm) and wgSNP distances. The cutoff for ANI was established at 95%; isolates exhibiting at least this percentage were classified as belonging to the same species (Figure 3) [28].

Based on this genomic evidence, isolates ATP111 and ATP210 were identified as *L. argentoratensis* and *W. cibaria*, respectively. Comparative genomic analysis within the genus *Lactiplantibacillus* revealed that the genome of *L. argentoratensis* ATP111 was closely related to *L. argentoratensis* DSM16365, with ANIb and ANIm exceeding 99% and exhibiting the lowest number of wgSNP distances (203 bp). *W. cibaria* ATP210 showed high genomic relatedness to the type strain *W. cibaria* JCM 12495, with ANIb and ANIm values exceeding 98% and a minimal pairwise wgSNP distance of 43 bp (Figure 3).

### Absence of AMR and virulence genes

Screening of the genomes of the two selected strains, ATP111 and ATP210, against four *AMR* gene databases (NCBI Bacterial AMR Reference Gene Database, CARD, ResFinder, and ARG-ANNOT) did not reveal any genes associated with AMR. Similarly, no virulence genes were identified in the genomes of isolates ATP111 and ATP210 through analysis using the VFDB.

### Secondary metabolite biosynthetic gene clusters

**Figure 4:**
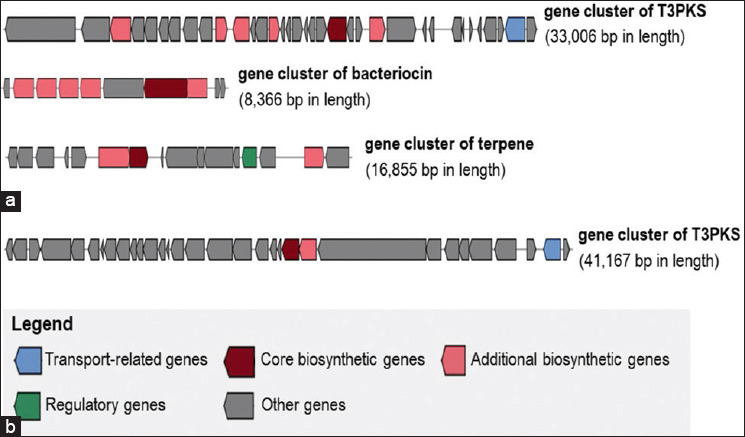
Clusters of putative secondary metabolite biosynthesis genes identified in the *ATP111* and *ATP210* genomes. (a) Three gene clusters associated with T3PKS, bacteriocin, and terpene biosynthesis detected in *Lactiplantibacillus argentoratensis* ATP111. (b) A single gene cluster associated with T3PKS biosynthesis in *Weissella cibaria* ATP210.

AntiSMASH analysis revealed secondary metabolite biosynthetic gene clusters in the genomes of *L. argentoratensis* ATP111 and *W. cibaria* ATP210. Three gene clusters were identified in *L. argentoratensis* ATP111, corresponding to type III polyketide synthases (T3PKS), bacteriocin, and terpene biosynthesis (Figure 4a). Cluster sizes ranged from 8.3 to 33.0 Kbp. The *T3PKS* gene cluster contained a core biosynthetic gene likely encoding hydroxymethylglutaryl coenzyme A synthase (HMG-CoA synthase). The core biosynthetic genes for the bacteriocin and terpene clusters were identified as a putative lactococcin-G-processing and transport ATP-binding protein and a putative 4,4′-diapophytoene synthase, respectively.

A single gene cluster associated with T3PKS biosynthesis was detected in the *W. cibaria* ATP210 genome, spanning 41.1 Kbp (Figure 4b). This cluster also contained a core biosynthetic gene encoding HMG-CoA synthase.

### Genomic basis for stress response in isolates ATP111 and ATP210

The gastrointestinal tract presents a challenging environment for microbial survival; however, the ability to adapt and survive under various stressors, including acidic pH, temperature fluctuations, bile salts, osmotic pressure, and oxidative stresses, is crucial for survival and subsequent LAB colonization [32].

**Figure 5:**
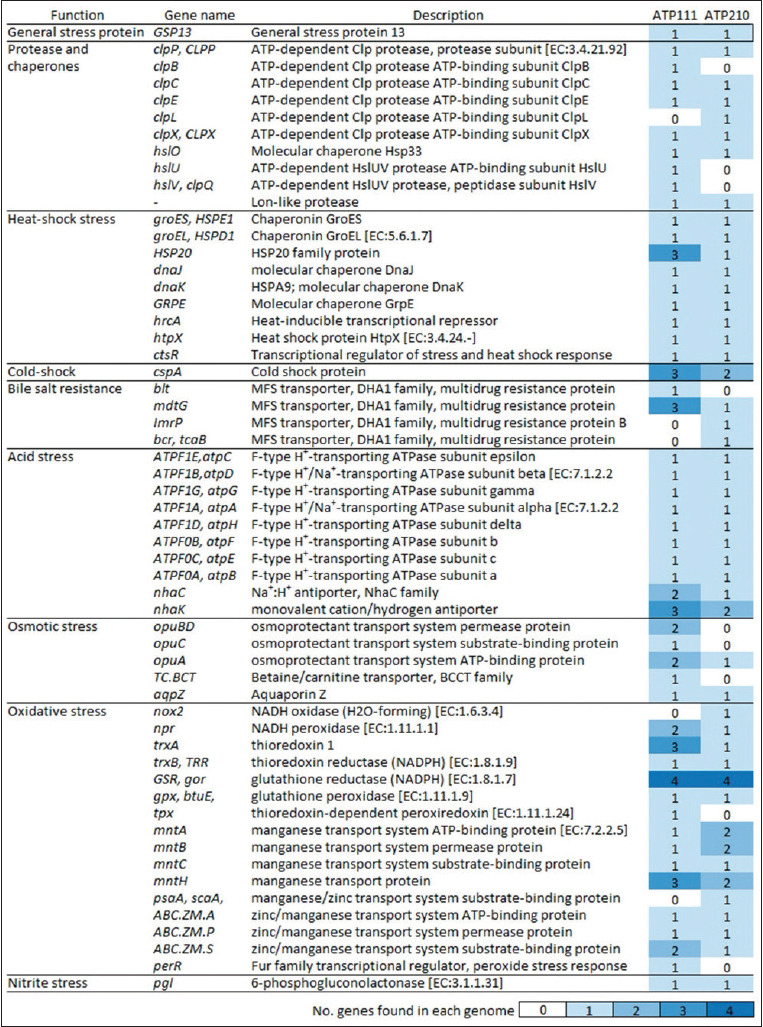
Distribution of stress response genes identified in the genomes of ATP111 and ATP210 isolates.

Genomic analysis of isolates ATP111 and ATP210 revealed the presence of several genes encoding proteins involved in stress response mechanisms, including genes associated with resistance to the aforementioned stress conditions encountered within the gastrointestinal tract (Figure 5 and Supplementary Table S1). Both isolates harbored core stress defense mechanisms, including general stress proteins, proteases, chaperones, and HSR elements. Notably, *L. argentoratensis* ATP111 exhibited a higher number of genes related to cold-shock response than *W. cibaria* ATP210, suggesting a potentially enhanced ability to withstand temperature fluctuations.

Both isolates possessed genes associated with bile salt resistance, which is crucial for survival in the small intestine, and genes involved in acid stress response, particularly F-type H^+^-transporting adenosine triphosphatase or ATP synthase, supporting their observed tolerance to low pH. *L. argentoratensis* ATP111 exhibited a greater number of genes related to osmotic stress response than *W. cibaria* ATP210, potentially indicating a higher adaptability to changes in osmolarity within the gastrointestinal environment. Both isolates contained multiple genes involved in combating oxidative stress, including those encoding NADH oxidase, thioredoxin, thioredoxin reductase, and glutathione reductase, highlighting their potential to neutralize reactive oxygen species. Genes associated with nitrite stress were also present in both strains.

These findings suggest that the identified stress response genes and their corresponding proteins likely contribute to the ability of ATP111 and ATP210 isolates to withstand the challenges of the gastrointestinal tract, supporting their resilience during *in vitro* assays. Moreover, the differences in the number of genes within specific categories may contribute to the variations in stress tolerance observed between the two isolates, which may be linked to the larger genome size of ATP111 compared with that of ATP210 (Table 3).

## DISCUSSION

### Strategic isolation and identification of autochthonous LAB from swine feces

In this study, the focus on evaluating the antibacterial activity and probiotic-related functional properties of autochthonous LAB isolated from swine feces through *in vitro* tests was a strategic approach to identify microorganisms that are potentially well adapted to the host’s gut environment. The selection of MRS medium for isolating LAB, particularly lactobacilli, was effective, yielding a significant number of presumptive isolates (64 out of 93). This outcome aligns with the established efficacy of MRS medium in inhibiting the growth of competing bacteria, such as *Bifidobacteria*, *Streptococcus*, *Enterococcus*, and fungi, as well as Gram-negative bacteria, thereby enriching LAB [33]. The subsequent confirmation of these isolates as Gram-positive, catalase-negative, and non-spore-forming bacteria provided a robust foundation for the *in vitro* assessment of their probiotic potential and antibacterial characteristics, enabling a targeted evaluation of their suitability for application in swine [34].

### Antibacterial activity and its functional relevance

Previous studies have consistently demonstrated the antimicrobial activity of LAB strains derived from various sources, including food [35] and animals, particularly swine [15]. Our findings further underscore the inherent capacity of LAB to inhibit the growth of various pathogens [36]. The observation that nine of our isolates exhibited inhibitory activity, with prominent broad-spectrum inhibition by isolates ATP111 and ATP210 against all pathogenic *E. coli* strains, aligns with these established reports and highlights the functional importance of antimicrobial activity as a desirable trait for probiotics. Notably, this *E. coli* panel consisted of key swine-specific pathotypes (EAEC, EHEC, EIEC, ETEC, and EPEC), underscoring the direct relevance of our findings to swine health and disease prevention. The antibacterial activities exhibited by LAB are commonly attributed to the production of a range of metabolic compounds, including organic acids such as lactic, acetic, and butyric acids, diacetyl, hydrogen peroxide (H_2_O_2_), carbon dioxide (CO_2_), fatty acids, ethanol, and bacteriocins [37, 38]. Our genomic analysis provides molecular insights into these potential mechanisms, revealing the presence of putative bacteriocin gene clusters in ATP111 and other secondary metabolite clusters in both isolates.

### Genomic insights into bacteriocin and secondary metabolite synthesis

Genomic analysis of *L. argentoratensis* ATP111 revealed a putative gene cluster encoding lactococcin-G, suggesting its potential to produce this bacteriocin. Lactococcin-G, a ribosomally synthesized, unmodified two-peptide (class IIb) bacteriocin known to form a membrane-penetrating helix–helix structure, has been reported in some LAB strains [39]. Its mechanism of action involves interaction with cell membrane receptors in susceptible bacteria, leading to cell membrane permeabilization and subsequent leakage of small monovalent cations [40]. In contrast, *W. cibaria* ATP210 lacked the crucial motifs for a bacteriocin gene cluster, a finding consistent with a previous genomic analysis of another *Weissella* strain that did not identify coding sequences for functional bacteriocin activity [41]. However, despite the absence of a predicted bacteriocin gene cluster, the observed antibacterial activity of *W. cibaria* ATP210 suggests the involvement of alternative inhibitory mechanisms. Indeed, *Weissella* species are recognized for producing diverse antimicrobial compounds, such as organic acids (e.g., lactic and acetic acids) and hydrogen peroxide that can collectively contribute to the suppression of pathogenic growth. Further investigation would be valuable to elucidate the specific compounds responsible for the inhibitory effects of ATP210. These genomic findings, including the presence of additional gene clusters (T3PKS and terpenes) in the ATP111 genome, are consistent with studies on other *Lactiplantibacillus* species, such as *L. plantarum*, which have highlighted the prevalence of gene clusters associated with various secondary metabolites, including bacteriocins, cyclic lactone autoinducers, terpenes, T3PKS, and ribosomally synthesized and post-translationally modified peptides [42, 43]. These genomic features suggest that *L. argentoratensis* ATP111 has a multifaceted potential to exert beneficial effects; however, further investigation is warranted to fully elucidate the expression and function of these gene clusters. To confirm the secretion of antimicrobial compounds, including putative bacteriocins, the potential inhibitory activity of cell-free supernatant (CFS) derived from *L. argentoratensis* ATP111 and *W. cibaria* ATP210 cultures against a panel of pathogenic *E. coli* strains and other relevant swine pathogens, such as *Salmonella* and *Clostridium* spp., should be investigated further.

### Acid and bile salt tolerance: Phenotypic and genomic correlation

The ability to withstand acidic conditions of the stomach (pH 2.0–2.5 in swine) and alkaline conditions imposed by bile in the duodenum is a critical prerequisite for the use of probiotics in swine feed supplements [15]. The gastric pH of sows and adult pigs is relatively low, typically ranging from 2.5 to 4 [44, 45]. Therefore, to simulate the swine gastric environment, an acid tolerance test was conducted at the lowest representative pH value of 2.5. The mean bile salt concentration in the intestinal environment of pigs has been reported to be 28.33 ± 9.56 mM, which corresponds to approximately 1% (w/v), and is considered physiologically relevant for *in vitro* tolerance testing of LAB [46]. In our study, while *L. argentoratensis* ATP111 and *W. cibaria* ATP210 exhibited a reduction in growth at pH 2.5 and in the presence of bile salts, indicating a degree of stress; they demonstrated survival, suggesting a level of tolerance to these conditions. These findings are consistent with observations from other studies on gut-isolated probiotics, which have reported varying levels of acid and bile salt tolerance [21].

The capacity of LAB to tolerate acid environments is related to several mechanisms, including the maintenance of cell membrane integrity, intracellular pH homeostasis, stabilization of vital biomolecules, and the activity of H^+^-ATPase [47, 48]. Genomic analysis of ATP111 and ATP210 revealed the presence of genes encoding a complete membrane-bounds F_0_F_1_-ATPase system (F-type H^+^-transporting ATPase) (atpC, atpD, atpG, atpA, atpH, atpF, atpE, and atpB) which consists of F_0_ (the membrane-integrated ion-translocating complex) and F_1_ (the peripheral catalytic complex) and a Na^+^/H^+^ antiporter (nhaC), which contribute to intracellular pH regulation through proton pumping [49]. Although bile salt hydrolase (BSH) activity has been associated with bile salt tolerance and persistence in LAB strains [50], neither ATP111 nor ATP210 encodes the Bsh protein. However, the presence of several genes encoding MDR transporters belonging to the MFS transporter family (blt and mdtG in ATP111 and ImrP, mdtG, bcr, and tcaB in ATP210) suggests a potential alternative mechanism for bile salt tolerance in these isolates, possibly through the efflux of bile salts [51].

The gastrointestinal tract also presents challenges related to temperature fluctuations, osmotic pressure, and oxidative stress [32]. The genomes of ATP111 and ATP210 harbored genes encoding proteins involved in adaptive stress responses, including general stress protein 13 (*GSP13*) and ATP-dependent Clp proteases (clpP, clpE, and clpX [ATP-dependent Clp protease]). Furthermore, genes encoding heat-shock proteins (grpE, hrcA, and *HSP20*), cold-shock proteins (cspA), chaperones (groES, *HSPE1*, groEL, *HSPD1*, hslO, dnaJ, and dnaK), an ATPase component (opuA), and enzymes involved in combating oxidative stress [NADH peroxidase (npr), thioredoxin (trxA and trxB), glutathione reductase, and manganese/zinc transport proteins (mntA, mntB, mntC, and mntH)] have been identified [49, 52, 53]. The presence of this diverse array of stress response genes likely contributes to the overall resilience of ATP111 and ATP210 observed during *in vitro* assays simulating the gastrointestinal environment. Although the genomic data are highly suggestive of probiotic potential, these claims should be considered as preliminary evidence. Further studies, such as functional assays (e.g., CFS inhibition tests and gene expression analysis under stress), are essential for validating these genomic findings.

### Adhesion capability and surface hydrophobicity

Adhesion to the intestinal mucosa is a critical selection criterion for probiotic microorganisms, facilitating intimate host-microbe interactions [54] and potentially supporting the competitive exclusion of pathogens [55]. This adhesion property serves as an indicator of the ability of a probiotic to attach to enterocytes [56]. The *in vitro* adhesion capability of the selected isolates to the glass test tube surface (a hydrophobic surface) was quantified by measuring the absorbance of bound crystal violet at 570 nm. A wavelength of 570 nm is commonly used to quantify the cell adhesion of LAB stained with crystal violet because the maximum absorbance (λmax) of crystal violet dissolved in ethanol or methanol is approximately 570 nm [57, 58]. From the results, ATP210 demonstrated higher adhesion than ATP111. This observation is consistent with previous reports indicating that LAB strains isolated from swine exhibit CSH associated with adhesive properties [59, 60]. The CSH of probiotic strains plays a significant role in their binding, colonization, and overall adhesion abilities [61], contributing to their interaction with both biotic (epithelial cells) and abiotic hydrophobic surfaces such as glass, stainless steel, and plastics [21, 62]. A higher degree of hydrophobicity typically indicates the presence of hydrophobic molecules on the bacterial cell surface, including surface array proteins, wall-intercalated proteins, cytoplasmic membrane proteins, and lipids [59, 60]. Although these *in vitro* adhesion results are promising, future investigations are warranted to assess their adhesive capabilities to relevant host epithelial cell lines and *in vivo* in swine models to elucidate their colonization potential fully.

### Safety assessment of the selected LAB isolates

The absence of β-hemolytic activity on blood agar is a crucial safety criterion for selecting probiotic candidates [63]. In this study, both *L. argentoratensis* ATP111 and *W. cibaria* ATP210 exhibited α-hemolytic activity. While β-hemolysis is typically associated with pathogenic potential, γ and α-hemolysis are regarded as safe [64, 65]. Some LAB strains displaying α-hemolysis have also been reported as probiotics in previous studies [21, 66], suggesting that the safety implications of α-hemolysis may be strain-dependent. Beyond hemolytic activity, the absence of transferable antibiotic resistance and virulence genes is an essential safety consideration for probiotics [67]. Our genomic analysis revealed that the selected isolates, ATP111 and ATP210, did not harbor any genes associated with AMR and virulence, indicating a favorable safety profile in these critical aspects.

### Survival in simulated gastrointestinal tract conditions

A fundamental characteristic of effective probiotics is their ability to survive transit through the gastrointestinal tract of the host. The gastric pH in swine typically ranges from 1.0 to 4.0, presenting a significant acid challenge [68]. The presence of bile salts in the proximal small intestine further demands microbial resilience. Therefore, the ability of LAB to withstand acidic conditions in the stomach and the presence of bile salts in the proximal small intestine are crucial for their functionality as swine feed supplements. In this study, the selected isolates, ATP111 and ATP210, demonstrated survival rates exceeding 50% under simulated gastrointestinal conditions. This level of survival is consistent with findings from a previous investigation, in which similar low pH and bile salt tolerances observed in LAB strains within a swine-simulated gastrointestinal tract were deemed satisfactory for probiotic application [69]. These results support the established understanding that probiotics can endure the stresses imposed by the acidic environment of the stomach and the presence of bile salts by employing various mechanisms, including maintenance of cell membrane integrity and functionality, regulation of intracellular pH, activity of H^+^-ATPase, and mechanisms for bile salt excretion. Genomic analysis of ATP111 and ATP210 revealed the presence of genes encoding a complete F_0_F_1_-ATPase system and a Na^+^/H^+^ antiporter, crucial for intracellular pH regulation. Furthermore, the identification of MFS transporters suggests an efflux mechanism for bile salt tolerance, whereas a diverse array of other stress response genes (e.g., heat-shock proteins, cold-shock proteins, and oxidative stress enzymes) likely contributes to their overall resilience in the complex gastrointestinal tract environment.

### Novel probiotic potential and comparative insights

The LAB is recognized as a significant component of the swine intestine microbiota, often representing a predominant bacterial group throughout the animal’s lifespan, although shifts in composition can occur [70]. Consistent with our findings, previous studies have revealed the potential for long-term colonization of specific LAB species in the gastrointestinal tract of pigs [70]. While some studies have explored certain probiotic attributes of *W. cibaria* isolated from porcine feces, such as bacteriocin production [71], a comprehensive characterization of its probiotic properties, particularly its antibacterial activity, has remained limited. To the best of our knowledge, this study provides the first detailed account of *W. cibaria* isolated from swine feces, demonstrating both its *in vitro* probiotic properties and inhibitory activity against pathogenic *E. coli*. Regarding *L. argentoratensis*, this species has undergone taxonomic re-evaluation and was previously classified as *L. plantarum* subsp. *argentoratensis* based on genomic analyses [72]. Although *L. argentoratensis* exhibiting promising probiotic properties has been isolated from diverse sources, including ready-to-eat products [73], fermented tea leaves [74], and fermented fish [75], and an isolate from swine manure with NH_3_-degrading activity has been reported by Bajagain *et al*. [76], research focusing on the isolation and comprehensive characterization of the probiotic properties of *L. argentoratensis* isolated from swine feces remains unexplored. Therefore, this study presents a novel contribution by demonstrating the *in vitro* antibacterial activity and a range of probiotic traits for *L. argentoratensis* isolated from swine feces. Further research is warranted to validate these properties *in vivo*, specifically to investigate the tolerance of *L. argentoratensis* and *W. cibaria* to the gastrointestinal environment of swine and to confirm their viability and compatibility as a mixed culture without antagonistic interactions. In addition, further studies will investigate the protective ability of *L. argentoratensis* and *W. cibaria* against pathogenic *E. coli* and their capacity to modulate the immune response in swine. The successful demonstration of these attributes *in vivo* would support the potential use of *L. argentoratensis* and *W. cibaria* as a mixed-probiotic feed additive for oral administration in swine.

### Relevance to the United Nations SDGs

The findings of this study contribute directly to several United Nations SDGs by addressing key aspects of animal health, AMR mitigation, and sustainable livestock production. The isolation of autochthonous *L. argentoratensis* and *W. cibaria* from swine feces, coupled with their demonstrated probiotic potential and absence of AMR or virulence determinants, supports SDG 3 (good health and well-being) by promoting alternatives to antibiotics that safeguard both animal and human health through the One Health framework. By enhancing gut health and productivity in pigs without relying on antibiotic growth promoters, these probiotics also align with SDG 2 (zero hunger) by contributing to improved food security and sustainable protein production. Furthermore, the replacement of antibiotic-based interventions with safe, host-adapted probiotic formulations addresses SDG 12 (responsible consumption and production) by reducing antimicrobial use and environmental contamination from resistant bacteria and residues. The use of microbiota-friendly, eco-compatible microbial feed additives additionally supports SDG 15 (life on land) by encouraging sustainable farming practices that preserve biodiversity and minimize ecological disruption. Overall, the development and application of host-derived probiotic candidates such as *L. argentoratensis* ATP111 and *W. cibaria* ATP210 exemplify a practical pathway toward achieving sustainable, responsible, and health-oriented livestock production systems in line with the SDG agenda for 2030.

## CONCLUSION

This study successfully isolated and characterized autochthonous LAB from swine feces, emphasizing their potential as probiotic alternatives to antibiotics in livestock production. Among 64 presumptive LAB isolates, *L. argentoratensis* ATP111 and *W. cibaria* ATP210 exhibited the most promising broad-spectrum antibacterial activity against multiple pathogenic *E. coli* pathotypes (EAEC, EHEC, EIEC, ETEC, and EPEC). Both isolates demonstrated tolerance to low pH and bile salts, moderate adhesion ability, and survival under simulated gastrointestinal conditions, fulfilling critical criteria for probiotic selection. Genomic analysis further confirmed the absence of AMR and virulence genes, highlighting their biosafety. The presence of genes encoding stress response proteins, F_0_F_1_-ATPase, Na^+^/H^+^ antiporter systems, and multidrug efflux transporters indicates a molecular basis for acid and bile resistance. In addition, *L. argentoratensis* ATP111 harbored gene clusters for bacteriocin (lactococcin-G), terpene, and type III polyketide synthase biosynthesis, suggesting its potential for producing diverse antimicrobial compounds.

From a practical standpoint, these findings support the use of host-adapted LAB as eco-friendly feed additives to improve swine gut health, enhance disease resistance, and reduce antibiotic dependency in pig farming. The deployment of such probiotics can mitigate AMR risks and contribute to safer animal-derived food production. Moreover, the use of autochthonous strains isolated from healthy pigs ensures better gastrointestinal adaptability and colonization efficiency, offering a sustainable biotherapeutic approach aligned with industry and One Health priorities.

The strengths of this study lie in its comprehensive integration of phenotypic assays with whole-genome sequencing, enabling molecular correlation between probiotic functionality and genomic determinants. The use of diverse *E. coli* pathotypes of clinical relevance strengthens the translational value of the findings. However, limitations include the confinement of the study to *in vitro* assays and the absence of *in vivo* validation in swine models. Gene expression analyses under stress and metabolomic profiling of secreted antimicrobial compounds were also beyond the present scope.

Future investigations should focus on *in vivo* evaluation of *L. argentoratensis* ATP111 and *W. cibaria* ATP210 to assess their survivability, colonization, immunomodulatory effects, and growth-promoting efficacy in pigs. Additional studies on synergistic potential in mixed-culture formulations and stability under industrial feed processing conditions are also warranted. The isolation and evaluation of similar host-specific LAB strains from different production systems may further expand probiotic diversity for species-targeted applications.

In conclusion, this study provides the first comprehensive phenotypic and genomic characterization of *L. argentoratensis* and *W. cibaria* isolated from swine feces, identifying them as safe and functionally robust probiotic candidates with broad-spectrum anti-*E. coli* activity. Their demonstrated biosafety, genomic stability, and stress resilience underline their suitability as sustainable alternatives to antibiotic growth promoters in swine production. These findings advance the goals of antimicrobial stewardship and sustainable livestock development in accordance with the United Nations SDGs (2, 3, 12, and 15), paving the way for the next generation of precision-designed probiotics in One Health-oriented animal agriculture.

## DATA AVAILABILITY

Raw Illumina reads were submitted to the National Center for Biotechnology Information SRA under BioProject accession number PRJNA957133. The assembled and annotated genomes of *L. argentoratensis* ATP111 and *W. cibaria* ATP210 were submitted to the National Center for Biotechnology Information under accession numbers JARXZI000000000 and JARXZH000000000, respectively. The data supporting the findings of this study are available from the corresponding author upon reasonable request.

## AUTHORS’ CONTRIBUTIONS

RJ and WC: First authorship, conceptualization, research design, methodology, data analysis, investigation, data curation, visualization, and writing of the original draft. RT, WP, WM, JK, and NS: Data analysis. RJ, WC, SC, WP, RT, WM, JK, NS, and SR: Reviewed and edited the manuscript. SR: Methodology, conceptualization, validation, supervision, and project administration. All authors have read and approved the final manuscript.
